# Opening of Two New Buildings

**Published:** 1920-07-31

**Authors:** 


### LEICESTER ROYAL INFIRMARY.

### Opening of Two New Buildings.

Thursday, July 22, was a unique occasion in the history of the above institution; not only did it mark the completion of two important and far-reaching developments—the work of the present Board and its Chairman—but it brought together a gathering of several hundred subscribers and friends, including four generations of the Chairman's family. The two buildings that were opened were the new opthopædic department and the new pathological laboratories, the architects being Messrs. Everard, Pick and Keay. Sir Arthur Grey Hazlerigg, Bart., presided, an incident that recalled the long association of his grandfather, Sir Arthur G. Hazlerigg, Bart., as Chairman of the Board.

### Leicestershire Freemasons' Gift.

In submitting a statement of the financial position relative to the orthopædic department, Mr. T. Fielding Johnson, Junr., Chairman of the Board, recalled a gift of £5,000 from the Freemasons of Leicestershire, which brought the building into being. The building would always be associated with Freemasons, as maintaining the highest traditions of their ancient Brotherhood. The original gift, however, was inadequate to meet the heavy cost of building, but with sums of £2,000 received from the Ministry of Pensions, and other amounts, all but £2,000 of the cost had been raised. He thought, however, it was desirable that the War Memorial to Freemasons fallen in the war should be opened free of debt, and subsequently a cheque would be presented to the Treasurer freeing the department from capital liability.

### A Unique Family Association.

The presiding Chairman then intimated that the youngest lady of the audience would present a cheque to the Treasurer. This was the youngest grand-daughter of the Chairman of the Board, a six-months-old little child, Miss Elizabeth Everard, who made the fourth generation of the family present, including the venerable father of the Chairman of the Board, himself Chairman of the institution some twenty years since and now in his ninety-second year, a family association unique in the memory of any present. Dr. Astley V. Clarke, having indicated the nature and importance of the work performed in the department, and the architect having presented a silver key, the new building was opened by the High Sheriff.

### £5,000 to Free Pathological Laboratories.

At the beginning of the war it was estimated that a sum of £5,000 would provide the new pathological laboratories, but the cost has more than doubled, and £5,000 is still required to meet the additions. The Mayor called attention to three important causes which made the extension of the institution necessary. First, the growth of the city and county; secondly, the growth of knowledge, particularly in medicine and surgery; and thirdly, the growth of the spirit of philanthropy. Dr. W. W. Mackarell, pathologist, having spoken on the work done in the pathological laboratories, paid tribute to the work of Dr. C. J. Bond, C.M.G., and the late Dr. T. Arnold Johnston, pioneer pathologist of the institution, who had done remarkable work under conditions very different from those that obtained at the present time.

The buildings were then formally opened, and the guests were entertained to tea by the Chairman of the Board and Mrs. Fielding Johnson. The band of the Desford Industrial School (from which patients are often received in the institution) played selections while the guests were assembling and during the close of the afternoon.

### A Story of Development and Progress.

At the half-yearly meeting of the Governors, held the previous day, a continuous note of progress was sounded. The buildings just described form only a part of the story of the development of the institution, centring round its third jubilee. A new south-west wing, to contain one hundred beds, will be commenced shortly, the money for the erection, for all practical purposes, having been raised. The addition of these hundred beds necessitates further accommodation for the nursing staff, and sixty bedrooms are in course of erection by two extensions of the nurses' home. The building is being pushed along speedily, and it is hoped to get the roofs on before the winter sets in.

### A Country Home of Recovery.

It was stated at the Governors' meeting that the infirmary had acquired the Leicester Frith Home, which Sir Jonathan North and his Soldiers' Fund Committee had placed at the disposal of the Board, with the equipment, subject to the Corporation's approval, as freeholders, on the understanding that the home should be used as an auxiliary institution for the reception of early convalescent patients. The home would contain accommodation for sixty patients.

There are also under consideration by the Board other important developments and new features of service, including the founding of a home for paying patients.

### Financial Statement.—Growing Income and Expenditure.

The statistical report on the work during the half-year shows a considerable increase in numbers in all the departments. One hundred and seven more civilian in-patients have been admitted than during the corresponding period of the previous year, the number totalling 2,237. There was a remarkable increase in the number of out-patients. The income for the six months was fairly satisfactory, the total being £21,893—£4,220 more than in the corresponding period of the previous year, due to an increase of about £1,700 in donations and £2,300 in the Saturday Hospital Society contributions, the employees having agreed to double their contributions to 2d. per week. The expenditure totalled £32,128. Provisions showed an increase of £1,200 on the corresponding period of last year; surgery and dispensary, an increase of £2,100, due to substantial purchases; domestic charges, an increase of £2,852, due to additional cost of coal, lighting, etc., and to purchases of bedding, etc. Salaries and wages had increased by £2,050, and management showed an increase of £400, the total increase of the expenditure on the corresponding expenditure of last year being 45 per cent. £3,450 had been received on account of legacies (£2,700 for the endowment of beds), and the balance of £750 was invested in the purchase of property to increase site area.

### Generous and Interested Residents.

A great feature in support of the institution was the enthusiastic interest shown by county parishes. Parades, galas, whist-drives, etc., had been organised, and the total result was not only gratifying to the Board, but spoke of the generosity of residents in districts far from the institution. It was no uncommon thing for £80 or £100 to be sent in from a village which had but a few houses, and the Board could not be too grateful to those who took up the organisation of those and similar efforts. The Board hoped that expenses had now reached the high-watermark, and they were encouraged by the support now being given to the institution. They hoped that the extensions that were in progress and under contemplation would not only be provided, but would be maintained by the addition of new contributors and new sources of revenue.

